# (Bio)polymer/ZnO Nanocomposites for Packaging Applications: A Review of Gas Barrier and Mechanical Properties

**DOI:** 10.3390/nano9101494

**Published:** 2019-10-19

**Authors:** Mohsin Abbas, Mieke Buntinx, Wim Deferme, Roos Peeters

**Affiliations:** 1 Packaging Technology Center, IMO-IMOMEC, Hasselt University, Wetenschapspark 27, 3590 Diepenbeek, Belgium; mohsin.abbas@uhasselt.be (M.A.); mieke.buntinx@uhasselt.be (M.B.); roos.peeters@uhasselt.be (R.P.); 2 Functional Materials Engineering Group, IMO-IMOMEC, Hasselt University, Wetenschapspark 1, 3590 Diepenbeek, Belgium; wim.deferme@uhasselt.be; 3 IMEC vzw-Division IMOMEC, Wetenschapspark 1, 3590 Diepenbeek, Belgium

**Keywords:** ZnO nanoparticles, (bio)polymer nanocomposites, packaging applications, barrier properties, mechanical properties

## Abstract

Nanotechnology is playing a pivotal role in improving quality of life due to its versatile applications in many areas of research. In this regard, nanoparticles have gained significant importance. Zinc oxide nanoparticles (ZnO NPs) amongst other nanoparticles are being used in producing nanocomposites. Methods like solvent casting, solution casting, solvent volatilization, twin-screw extrusion, melt compounding and extrusion blow molding have been applied to produce ZnO NPs based (bio)polymer composites. These composites are of great interest in the research area of food packaging materials due to their improved multifunctional characteristics like their mechanical, barrier and antimicrobial properties. This paper gives an overview of the main methods to synthesize ZnO NPs, methods to incorporate ZnO NPs in (bio)polymers, and finally, the gas barrier and mechanical properties of the nanocomposites. As a conclusion, a maximum decline in oxygen, carbon dioxide and water vapor permeability was reported as 66%, 17% and 38% respectively, while tensile strength and young’s modulus were observed to increase by 32% and 57% respectively, for different (bio)polymer/ZnO nanocomposites.

## 1. Introduction

Nanotechnology combined with other areas of research such as the life sciences, physics, chemistry, medicine, engineering, the cognitive sciences, and information technology has gained significant importance due to its broad applications [1]. It has also proven its significance in the field of packaging technology. Its applications have also become a good basis to bring large benefits to the food and nutrition sector [2,3,4]. In recent years, nanotechnology has emerged as a multibillion-dollar global industry expected to surpass US $125 billion mark by 2024 [5]. The world’s leading food companies like Nestle, Unilever, Kraft, and Heinz are exploring nanotechnology in food processing and packaging areas [6]. Food packaging materials implementing nanotechnology are the largest category of application of nanotechnology for the food sector [7]. Smart and intelligent packaging systems, the use of biosensors in bacteria identification, and monitoring of food quality are some of the emerging applications of nanotechnology [8]. Food is a necessary part of our daily life, which has to be protected from undesirable factors that can potentially decrease its nutritional value and shelf-life. Depending on the food to be preserved, packaging requires specific moisture, gas and/or antimicrobial barriers. Food packaging is the sole consumer of more than 20% of the plastics produced [9]. However, the mainstream of plastic packaging materials has originated from petroleum resources. Non-sustainability and non-biodegradability are two important issues related to these plastic materials. Poor barrier properties against moisture and gases are also severe issues in food packaging as food products need optimal conditions to preserve them. Biopolymers are environmentally friendly materials and have the potential to improve food protection while retaining its quality and safety. These materials have also mitigated the environmental issues that are related to conventional polymers [10]. However, the main disadvantages associated with these materials are their weak barriers and mechanical properties which make them unfavorable for many food packaging applications [11]. Recent developments in (bio)polymer nanocomposites have assisted in expanding their functional properties [12]. Extensive research and development activities both in academia and industry can be helpful to cope with the above-mentioned challenges.

A variety of nanoparticles (NPs) reinforced composites have been developed that typically consist of up to 5% w/w NPs [13]. The addition of NPs into films has made end-products fire resistant, lightweight, stronger in thermal and mechanical performance and less permeable to moisture and gases. When incorporated in plastic films, nanoparticles-such as zinc oxide (ZnO) and silica and titanium dioxide (TiO_2_) are capable of reducing the flow of oxygen inside packaging containers. They also act as a barrier against moisture to keep food fresh for an extended period [14]. Silicon dioxide (SiO_2_) and TiO_2_ are the most commonly used nanoparticles in food packaging. SiO_2_ is used as a drying and anticaking agent [15]. TiO_2_ can be used as a barrier against UV protection for food packaging. These nanoparticles have also been reported for their antimicrobial activity, however, their applications are limited due to their photocatalytic behavior [16]. Silver nanoparticles can protect food from microbial invasion [17]. Copper and copper oxide, cadmium, ZnO, telluride, magnesium oxide, single-walled carbon nanotubes are also reported for their antimicrobial activity [18].

Nanomaterials in the field of food packaging are creating a tremendous impact by improving the numerous properties of packaging films. Zinc oxide nanoparticles (ZnO NPs) amongst other nanoparticles have gained a vital position in the enhancement of packaging properties like barrier, mechanical and antimicrobial properties [19]. These ZnO NPs consisting of various particle sizes and shapes have been synthesized via different routes. Their availability at commercial scale has made their access easy to use them directly in research experiments. Packaging films consisting of ZnO NPs along with (bio)polymers have been made through diverse processing techniques, such as solvent casting [20,21], melt compounding [22], solution casting [23,24,25,26], twin-screw extrusion [27], extrusion blow molding [28], and solvent volatilizing [29].

This article reviews the methods used to synthesize ZnO NPs and the methods to incorporate them in (bio)polymers, and then gives an overview of the use of ZnO NPs in packaging materials in order to improve their gas barrier and mechanical properties.

## 2. Zinc Oxide Nanoparticles

### 2.1. Synthesis Methods of ZnO NPs

A variety of synthesis methods to produce ZnO NPs have been published worldwide, e.g., sol-gel process, hydrothermal process, wet chemical method, etc. Different orientations, morphologies, size, and shapes of ZnO NPs can be obtained by varying a set of parameters such as type of solvent, reaction temperature, etc. Some important categories of synthesis methods of ZnO NPs are briefly described here.

The sol-gel method is a widely used method by scientists to synthesize ZnO NPs [30,31,32,33,34,35,36,37,38,39]. The sol-gel method is based on colloidal chemistry. Sols are referred to as the colloidal solution that consists of solid particles suspended in a liquid phase. Gels are typically formed by the polycondensation or polyesterification methods followed by aging to achieve phase transformations. This transformation is a very important step in the development of particle formation [40].

Among other methods, this technique has high reliability, low production cost, good repeatability, low process temperature, simplicity of the process, ease of control on morphology of nanoparticles and good homogeneity [41,42].

The hydrothermal method requires no organic solvents. The autoclave is used in the synthesis process, where a mixture of substrates is gradually heated to a temperature of 100–300 °C and then left for several days. As a result, crystal nuclei with nano dimensions are formed. Depending on the process conditions and materials used, crystals with a variety of shapes and dimensions can be obtained [43,44].

Sonochemical technology has gained significant attention in the synthesis of different compounds in material science. It is based on acoustic cavitation which results from the continuous formation, growth and implosive collapse of bubbles in a liquid [45]. Some researchers have also used this method to prepare different shapes of ZnO nanocrystallites [46].

The microemulsion method can be defined as an isotropic, thermodynamically stable system constituting smaller droplets dispersed in an immiscible solvent and an amphiphilic surfactant species on the surface of the micelle. For self-assembly and size-selective preparation of nanoparticles, the microemulsion mediated synthesis has been used due to excellent control on the particle size [47,48,49,50].

The solvothermal method is an appropriate method for the preparation of both crystalline oxide and non-oxide materials. Crystalline solids, including silicate materials with high porosity like zeolites [51] and oxide or non-oxide nanoparticles [52] can be produced by this method. The main advantage of this method is that the problems like solvents’ toxicity and the inability of dissolving the salts can be overtaken [53].

The chemical vapor deposition (CVD) method involves the deposition of a solid film on a heated surface by a chemical reaction from the gas or vapor phase. Different types of CVDs include plasma CVD, photo-laser CVD, thermal CVD, metal-organic chemical vapor deposition (MOCVD), and laser CVD, etc. For example, in thermal CVD, a temperature above 900 °C is used to activate the reaction [54].

The co-precipitation method normally involves the reaction of inorganic alkalis with zinc (Zn) salt. Precipitates produced from this reaction are washed and then calcined. In this method, calcination is done at different temperatures in order to achieve the nanoparticles with required morphology [55].

The microwave-assisted synthesis method is clean, simple, and without the problems of thermal gradient effects [56]. This process is widely used to produce hydroxide, oxide, and sulfide nanoparticles, etc. [57].

The green synthesis method is an eco-friendly, energy-efficient and cost-effective approach to synthesize nanoparticles. Plants, bacteria, fungi, algae, and viruses are used to synthesize ZnO NPs from this method [58].

In general, the preparation of nanoparticles is a complex process that contains different variables in the chemical reactions. It is difficult to control all the variables in the synthesis process. Each of these variables might have an effect on the properties of the end product. The most important variables are the type of solvent and precursor as well as physical and chemical conditions like pH and temperature [59]. Table 1 represents an overview of some synthesis methods and the respective size and morphology of the obtained ZnO NPs.

### 2.2. Commercial Grade ZnO NPs

There are several companies manufacturing ZnO NPs with different particle sizes, shapes, purity and phase states. Commercial, highly purified ZnO NPs are available in the range from 10 to 800 nm and they exist in powder, aqueous, suspension, emulsion and dispersion forms. Table 2 covers a list of vendors of ZnO NPs along with the information about the characteristics of these nanoparticles.

## 3. Production of (Bio)Polymer/ZnO Nanocomposites

Many (bio)polymers such as linear low density polyethylene (LLDPE), poly(lactic acid) (PLA), chitosan, poly(3-hydroxybutyrate-co-3-hydroxyvalerate) (PHBV), poly(3-hydroxybutyrate) (PHB), poly(butylene adipate-co-terephthalate) (PBAT), low density polyethylene (LDPE), semolina flour and bovine skin gelatin type-B (BSG) have been incorporated with ZnO NPs with different particle shapes and sizes through different processing methods to produce (bio)polymer nanocomposites such as solvent casting, solution casting, melt processing, and extrusion process, etc.

The solvent casting process contains ultrasonication or vigorous stirring of the nanoparticles in a polymer solution before casting in a mold and then evaporation of the solvent. Both organic solvents and water can be used to produce nanocomposites with either thermosets or thermoplastics [88]. In solution casting, a polymer is dissolved in a suitable solvent and then the active compound of interest is incorporated, then this mixed solution is poured on an inert surface. Upon solvent evaporation, films with the desired functionalities are formed [89]. In an extrusion process, the active compound and polymers are melted in a single or twin-screw extruder by heat transfer that leads to a blend formation from which films can be obtained [88]. Melt compounding or melt blending is a simple and cost-effective technique that is free from using toxic solvents. Blending can be done by using extrusion or injection molding methods [90].

Table 3 provides the information about ZnO NPs, (bio)polymers, characteristics and their manufacturers; and the processing methods to develop ZnO NPs based (bio)polymer composites.

## 4. Barrier Properties of (Bio)Polymer/ZnO Nanocomposites

Nanocomposites can enhance barrier properties by providing multipurpose chemical functionality. Significant improvement in the barrier properties on the incorporation of nanoparticles into the polymers has been reported [95,96,97,98]. The oxygen transmission rate (OTR) is defined as the amount of oxygen gas passing through an area in a certain time under specified conditions of temperature, humidity and pressure and its units are cm^3^/(m^2^.day) [99]. Permeability coefficients of oxygen (PO_2_-cm^3^.mm/(m^2^.day.atm)) and carbon dioxide (PCO_2_-cm^3^.mm/(m^2^.day.atm)) are obtained by multiplying the OTR and CO_2_-TR with the thickness (mm) of the sample, respectively [100]. While the water vapor transmission rate (WVTR) is the quantity of water vapor transmitted through an area in a certain time under specified conditions of humidity and temperature and it is presented in g/(m^2^.day) [101]. Water vapor permeability (WVP-g.mm/(m^2^.day)) is the product of permeance and thickness (mm) of the sample [102].

Jasim et al. [28] reported that the OTR has decreased by 23.2% upon 10 wt% ZnO NPs loading for LLDPE films reinforced with ZnO NPs. A uniform distribution of ZnO NPs in the polymer matrix could contribute to this decrease in OTR. For a PLA/ZnO bionanocomposite, an 18% and 17% decrease in PO_2_ and PCO_2_ were shown, respectively, due to the uniform distribution of ZnO nanoparticles in the PLA matrix, while WVP was increased by 16% [27].

For ZnO NPs based biocomposites, on the addition of 4 wt% ZnO in PHBV, the PO_2_ value was observed to decrease up to 35% due to strong interfacial adhesion with the polyester matrix, which caused chain immobilization [23]. In the case of ZnO/PHB bionanocomposites, upon 5 wt% ZnO NPs loading-PO_2_ value was decreased by about 53% and a decreasing trend was also observed for WVP value by up to 38% for the same ZnO NPs loading [24]. For ZnO/PBAT composite, there was a decreasing trend in OTR values upon increasing ZnO NPs loading range from 0 to 10 wt%. The lowest value of OTR was observed for 10 wt% ZnO NPs loading [25].

In a research conducted on untreated and 3-methacryloxypropyltrimethoxysilane treated ZnO nanoparticle reinforced-PLA nanocomposites, PO_2_ values of plasticized PLA film decreased by 36.07% and 55.1% by adding 10% ZnO (untreated) and ZnO (3-methacryloxypropyltrimethoxysilane treated) NPs, respectively [20]. This enormous decrease in PO_2_ values could be ascribed to the reinforcement of ZnO NPs with its high aspect ratio and its nice distribution throughout the polymer matrix [103].

In a study on Semolina reinforced with nanofillers (ZnO-nanorod/nano-kaolin), PO_2_ was decreased by up to 66% [21]. Wenhui Li et al. [29] reported in their research on ZnO based PLA nanocomposite that OTR was decreased on increasing ZnO content. The tortuous pathway prolongs the oxygen pathway and is the main reason for the improvement of oxygen resistance in the nano-blend films [104]. However, there was an increasing trend for WVP that could be due to the hydrophilicity of nano-ZnO and the improved hydrophilic interaction of the films.

For bovine skin gelatin type-B (BSG) composite films incorporated with ZnO nanorods and clove essential oil (CEO), PO_2_ was decreased by 32.27% by the addition of 2 wt% into the polymer matrix. This reduction in PO_2_ value might be due to the uniform dispersion and high aspect ratio of ZnO nanorods into the gelatin matrix [26].

It can be concluded from permeability tests of ZnO NPs based (bio)polymer composites that the maximum decrease in PO_2_ value by up to 66% is observed for semolina reinforced with nanofillers [21] where ZnO particles have been used in nanorods shape. In addition, a 17% decrease in PCO_2_ upon the incorporation of 1 wt% ZnO in ZnO/PLA biocomposites [27] and 38% decrease in WVP value at 5 wt% ZnO NPs loading in ZnO/PHB nanobiocomposites [24] are reported.

In summary, the barrier properties of the nanocomposites can be improved by a high aspect ratio, uniform dispersion and low incorporation (up to 5 wt%) of nanoparticles within the polymer matrix. 

The barrier properties regarding various ZnO NPs based (bio)polymer composites are represented in Table 4.

## 5. Mechanical Properties of (Bio)Polymer/ZnO Nanocomposites

Incorporation of ZnO NPs into the polymer matrix has shown a significant effect on the mechanical properties of the composites, which include tensile strength, impact strength, young’s modulus, stress at yield, strain at break, elastic strength and elongation at break.

Roberto Pantani et al. [22] reported that PLA-ZnO nanocomposite films showed a slight increase in Young’s modulus and low elongation at break in tensile tests. The addition of a plasticizer into PLA-ZnO nanocomposite films could be considered for the improvement of these parameters. Tensile tests on ZnO/PHBV nanobiocomposites showed that Young’s modulus was increased by 57% on 4 wt% ZnO NPs loading. An increase in the crystallinity of PHBV, homogeneous distribution of nanoparticles and strong interfacial adhesion between the phases could be the possibilities for the significant increase in Young’s modulus. However, the strain at break was decreased by 30% with increasing ZnO NPs content. Due to filler reinforcement, the ductile flow of the polymer chains was restricted, which was also depicted from low strain at break values [23].

In the case of ZnO/PHB nanobiocomposites, the tensile strength, young’s modulus, and impact strength were increased up to 32%, 43% and 26% respectively due to a strong interfacial adhesion between the matrix and the nanofiller via interactions by hydrogen bonding [24]. Tensile strength and elongation at break values were increased in the case of ZnO/PBAT nanocomposites. This could be due to the fine distribution of ZnO NPs in the polymer matrix [25]. Songee Beak et al. [92] reported the analysis of the tensile tests on an olive flounder bone gelatin-ZnO nanocomposite. They observed that the tensile strength of the nanocomposite films was increased while elongation at break was decreased by 37%. Intermolecular interactions of ZnO NPs with gelatin molecules were responsible for improving the rigidity of the gelatin films.

In a study on LDPE/ZnO nanocomposites, the values of elongation at break and tensile strength were decreased due to agglomeration and poor interfacial adhesion in the polymer nanocomposites [93]. A similar trend of decreasing elongation at break and tensile strength was observed for ZnO based PP nanocomposites [94].

It can be concluded from the above-mentioned analyses on mechanical properties of ZnO nanoparticles based (bio)polymer composites that a maximum increase in tensile strength up to 32% has been observed for ZnO/PHB bionanocomposites [24], while a 57% increase in young’s modulus upon 4 wt% ZnO loading in ZnO/PHBV bionanocomposites [23] is reported.

In summary, the uniform distribution of nanoparticles within the polymer matrix and strong interfacial adhesion can lead to enhanced mechanical properties of nanocomposites. The mechanical properties of ZnO nanoparticles based (bio)polymer composites are summarized in Table 5.

## 6. Conclusions

Nanotechnology is providing new possibilities for controlling properties, adding value and introducing new features in almost all scientific and technological fields to improve the quality of life. In this regard, nanocomposites are contributing significantly as they offer excellent opportunities to explore new functionalities as compared to conventional materials. Owing to the multifunctional properties in (bio)polymer composites, ZnO NPs have drawn interest of a lot of researchers in the diversified area of food packaging. The aim of this paper was to summarize the main methods to synthesize ZnO NPs, the methods to incorporate ZnO NPs in (bio)polymer matrices and the impact of the ZnO NPs incorporation on the gas barrier and mechanical properties of the (bio)polymer nanocomposites. These innovative (bio)polymer/ZnO nanocomposite films can be potentially applied as food packaging materials with improved properties. Based on comprehensive investigations on ZnO NPs based (bio)polymer nanocomposites, a well-defined feature of these nanocomposites is the small size of nanoparticles that leads to an increased interfacial area as compared to the traditional composites. The interfacial interaction between ZnO NPs and the polymer matrix plays an important role in improving the final properties. Upon surface modification of these nanoparticles, the desired properties of the matrix can be improved further even at lower wt% of nanoparticles. However, there are certain challenges associated with the production of nanocomposites such as: (1) a uniform dispersion of the nanoparticles in the matrix and (2) a proper selection of manufacturing techniques in order to regulate the level of dispersion of these nanoparticles which also create a significant impact on reinforcement as well as end-product properties. Moreover, tremendous research efforts are essential in moving towards the industrialization of ZnO/(bio)polymer nanocomposites to fulfill the requirements of numerous technological fields.

## Figures and Tables

**Table 1:** Overview of synthesis methods and morphology of zinc oxide (ZnO) nanoparticles (NPs).

Method	Materials	Size (nm)	Shape	Reference
Hydrothermal	Zinc acetate dihydrate, polyvinylpyrrolidone (PVP)	L: 5000, D: 50–200	Nanorods	[60]
	Zinc acetate dihydrate, zinc chloride, sodium hydroxide	60	Nanorods	[61]
Microwave decomposition	1-Butyl-3-methylimidazolium bis (trifluoromethylsulfonyl) imide [bmim][NTf2], zinc acetate dehydrate	37–47	Sphere	[62]
Co-precipitation	Zinc acetate, double distilled water	D: 30–60, L: 80	Nanorods	[63]
	Tetrahydrated zinc nitrate, ammonium hydroxide	20–40	Crystals	[64]
Micro-emulsion	Zinc acetate dihydrate, ethylbenzene acid sodium salt (EBS), xylene, dodecylbenzene sulfonic acid sodium salt (DBS), ethanol and hydrazine	D_DBS_: 300 D_EBS_: 80	Nanorods	[65]
	Zn(AOT)_2_, heptane, diethyl oxalate, chloroform, methanol	10–20	Quasispherical	[66]
Solvothermal	Zinc acetate dihydrate, polyethylene glycol, absolute ethanol	10–20	Quasispherical	[66]
	Triethanolamine, zinc acetylacetonate monohydrate, 1-octanol, and absolute ethanol	L_rod_ ~100 D_sphere_ ~20	Rods (ethanol without triethanolamine—TEA) spherical (ethanol with TEA)	[67]
Sol-gel	Oxalic acid dihydrate, zinc acetate dihydrate, hydrochloric acid, ammonia, and absolute ethanol	20	Spherical	[39]
Sonochemical	Potassium hydroxide, zinc nitrate hexahydrate, and cetyltrimethylammonium bromide	200–400 wide, a few nm thick	Flakes	[68]
Chemical vapor deposition	Zinc acetate dihydrate, ethanol	Average D: 90 and L: 564	Nanorods	[69]
Electrochemical	Oxalic acid dihydrate purified, Zn electrode, potassium chloride, nitric acid, and sodium hydroxide	D_spherical_: 50–100 L_cylindrical_: 150–200	Spherical and cylindrical particles	[70]

**Table 2:** Some of the major ZnO NPs vendors.

Sr. No.	Company	Physical Characteristics	Phase	Country	Reference
1	Meliorum Technologies Inc.	10 nm	Powder, aqueous, dispersion	United States	[71]
2	Sukgyung AT Co., Ltd.	10–20 nm, 20–40 nm	Powder	Korea, Republic	[72]
3	US Research Nanomaterials, Inc.	10–30, 18, 20, 35–45, 30–40, 50–80, 80–200 nm	Powder, dispersion	United States	[73]
4	SkySpring Nanomaterials, Inc.	10–30 nm, <30 nm, 200 nm, 200–800 nm	Powder	United States	[74]
5	Stanford Advanced Materials	17–27 nm, 30–50 nm, 70–90 nm	Powder	United States	[75]
6	Ultrananotech	≥20 nm	Powder	India	[76]
7	Advanced Nano Products	20–30 nm	Powder, emulsion	Korea, Republic	[77]
8	MKnano	20, 30, 40, 50–150 nm	Powder	Canada	[78]
9	Nanophase™ Technologies	20, 40, 60 nm (elongated)	Powder, dispersion	United States	[79]
10	Inframat^®^ Advanced Materials™	~30 nm	Powder	United States	[80]
11	TECNAN	30–40 nm	Powder	Spain	[81]
12	Micronisers	30–50 nm	Powder	Australia	[82]
13	EPRUI Nanoparticles & Microspheres Co. Ltd.	30, 50, 200 nm (nearly spherical)	Powder	China	[83]
14	American Elements	≤40 nm	Powder, dispersion	United States	[84]
15	Linari NanoTech	45 ± 5 nm	Suspension	Italy	[85]
16	Sigma Aldrich^®^	<50 nm, <100 nm, <110 nm, <130 nm	Powder, dispersion	United States	[86]
17	Nyacol^®^ Nanotechnologies, Inc.	50, 125 nm	Suspension	United States	[87]

**Table 3:** ZnO NPs, (bio)polymers and incorporation/deposition methods to produce ZnO NPs based (bio)polymer composites.

Characteristics-ZnO NPs	Supplier	Polymer	Biopolymer	Supplier	Incorporation Method	Reference
Particle size <100 nm	Sigma Aldrich (St. Louis, MO, USA)	Linear low density polyethylene (LLDPE)	-	Equate Petrochemical Co. (Kuwait)	Extrusion blowing method	[28]
Particles size 100–500 nm	Pylote SAS in Dremil-Lafage, France	-	Poly(lactic acid) (PLA)	NatureWorks LLC (USA)	Twin-screw extrusion	[27]
Rod-like particles, ZnO content: 96.2 ± 0.5%	Umicore, Belgium	-	PLA	NatureWorks	Melt compounding	[22]
Average particle size <25 nm	Sigma Aldrich	-	Chitosan	Sigma Aldrich	NA	[91]
Particle size <100 nm	Sigma Aldrich	-	Poly(3-hydroxybutyrate-co-3-hydroxyvalerate) (PHBV)	Goodfellow Corp.	Solution casting	[23]
Particle size <100 nm	Sigma Aldrich	-	Poly(3-hydroxybutyrate) (PHB)	Biomer Ltd. (Krailling, Germany)	Solution casting	[24]
Particle size 60 nm, spherical in shape	Co-precipitation method using zinc acetate and sodium hydroxide precursors	-	Poly(butylene adipate-co-terephthalate) (PBAT)	BASF Ltd., Japan	Solution casting	[25]
Nanopowder, <50 nm particle size	Sigma Aldrich Co. (St. Louis, MO, USA)	Olive flounder (Paralichthys olivaceus) bones	-	A seafood restaurant in Daejeon, Korea	NA	[92]
Particle size 30 nm	Umicore, Belgium	-	PLA Ingeo™ 4043D	NatureWorks LLC (Minnetonka, MN, USA)	Solvent casting	[20]
An average particle diameter of 70 nm	Pars Nanonasb (Persia)	Low density polyethylene (LDPE)	-	Petkim (Turkey)	Melt blending method	[93]
An average particle diameter of 70 nm	Pars Nanonasb (Persia)	Polypropylene (PP)	-	Borealis (Vienna, Austria)	Melt blending method	[94]
Nanorods	Catalyst-free combust oxidized mesh process	-	Semolina flour	Local market in Tehran, Iran	Solvent casting	[21]
Purity = 99.9%	MaiKun Industrial Co., Ltd. (Shanghai, China)	-	PLA	NatureWorks LLC (Lincoln, NE, USA)	Solvent volatilizing method	[29]
Particle size <100 nm	Umicore, Belgium	Bovine skin gelatin type-B (BSG)	-	Sigma-Aldrich (St. Louis, MO, USA)	Solution casting	[26]

**Table 4:** Barrier properties of ZnO NPs based (bio)polymer composites.

ZnO NPs Based Composites	Oxygen Transmission Rate (OTR)/Oxygen Permeability (PO_2_)	Water Vapor Permeability (WVP)	Carbon Dioxide Permeability (PCO_2_)	Reference
LLDPE films reinforced with ZnO NPs	OTR decreased by 23.2% for 10 wt% ZnO incorporation	NA	NA	[28]
PLA/ZnO biocomposite	For 1 wt% ZnO incorporation, oxygen permeability (PO_2_) decreased by 18%. Then there is no further decrease for addition up to 5 wt%	For 1 wt% ZnO incorporation, water vapor permeability (WVP) increased by 16%. Then there is no change for higher ZnO content.	For 1 wt% ZnO incorporation, carbon dioxide (CO_2_) permeability decreased by about 17%. Then there is no further decrease for higher ZnO content.	[27]
PLA-ZnO nanocomposite films	NA	WVP decreased on increasing ZnO NP concentration from 1 to 3 wt%	NA	[22]
Carboxymethyl cellulose-chitosan-ZnO NPs nanocomposites	NA	WVP decreased on increasing ZnO NP concentration up to 2 wt%	NA	[91]
ZnO-reinforced PHBV bionanocomposites	PO_2_ decreased up to 35% with 4 wt% ZnO loading	NA	NA	[23]
ZnO/PHB bionanocomposites	PO_2_ decreased by about 53% at 5 wt% ZnO NPs loading	WVP decreased by up to 38% at 5 wt% ZnO NPs loading	NA	[24]
ZnO/PBAT nanocomposite films	Lowest value of OTR observed for 10 wt% ZnO NPs loading (for 0-10 wt% ZnO NPs loading range)	NA	NA	[25]
An olive flounder bone gelatin-ZnO nanocomposite	NA	WVP decreased	NA	[92]
Untreated and 3-methacryloxypropyltrimethoxysilane treated ZnO nanoparticle reinforced-PLA nanocomposites	PO_2_ values of plasticized PLA film reduced by 36.07 and 55.1% with the incorporation of 10% ZnO (untreated) and ZnO (3-methacryloxypropyltrimethoxysilane treated) NPs	NA	NA	[20]
ZnO based LDPE nanocomposites	OTR decreased by 17% on adding 5 wt% ZnO NPs	WVTR decreased by 22% on adding 5 wt% ZnO NPs	NA	[93]
ZnO based PP nanocomposites	OTR decreased by 22% on adding 5 wt% ZnO NPs	WVTR decreased by 12% on adding 5 wt% ZnO NPs	NA	[94]
Semolina reinforced with nanofillers (ZnO-nanorod/nano-kaolin)	PO_2_ decreased by up to 66%	NA	NA	[21]
ZnO based PLA nanocomposite	Decreased	Increased	NA	[29]
Bovine skin gelatin type-B (BSG) composite films incorporated with ZnO nanorods and clove essential oil (CEO)	PO_2_ decreased by 32.27% with the addition of 2 wt% ZnO NPs	NA	NA	[26]

**Table 5:** Mechanical properties of ZnO NPs based (bio)polymer composites.

ZnO NPs Based Composites	Effect on Mechanical Properties	Reference
LLDPE films reinforced with ZnO NPs	Elongation at break: decreased; Tensile strength: increased	[28]
PLA/ZnO biocomposite	Stress at yield: higher in the machine direction; Elongation at break: higher in the machine direction	[27]
PLA-ZnO nanocomposite films	Young’s modulus: slightly increased; Elongation at break: decreased	[22]
ZnO-reinforced PHBV bionanocomposites	Young’s modulus: increased by ~57% on 4 wt% ZnO NPs loading; Strain at break: decreased by ~30% on increasing ZnO NPs content	[23]
ZnO/PHB bionanocomposites	Tensile strength: increased up to 32%; Young’s modulus: increased up to 43%; Impact strength: increased up to 26%	[24]
ZnO/PBAT nanocomposite films	Elongation at break: increased; Tensile strength: increased	[25]
An olive flounder bone gelatin-ZnO nanocomposite	Elongation at break: decreased by ~37%; Tensile strength: increased	[92]
Untreated and 3-methacryloxypropyltrimethoxysilane treated ZnO nanoparticle reinforced-PLA nanocomposites	Tensile strength: decreased in ZnO NPs (untreated) composites; increased in ZnO NPs (3-methacryloxypropyltrimethoxysilane treated) composites; Elongation at break: decreased in ZnO NPs (untreated) composites; a marginal drop in ZnO NPs (3-methacryloxypropyltrimethoxysilane treated) composites	[20]
ZnO based LDPE nanocomposites	Elongation at break: decreased; Tensile strength: decreased	[93]
ZnO based PP nanocomposites	Elongation at break: decreased; Tensile strength: decreased	[94]
ZnO based PLA nanocomposite	Elongation at break: increased; Tensile strength: decreased	[29]
Bovine skin gelatin type-B (BSG) composite films incorporated with ZnO nanorods and clove essential oil (CEO)	Elongation at break: increased; Tensile strength: increased	[26]
